# Effects of the Center-Edge Gradient and Habitat Type on the Spatial Distribution of Plant Species Richness in Santiago, Chile

**DOI:** 10.3390/plants14223433

**Published:** 2025-11-10

**Authors:** Sergio A. Castro, Cristian Ray, Javier A. Figueroa, Mathías Alfaro, Fabiola Orrego, Pablo M. Vergara

**Affiliations:** 1 Laboratorio de Ecología y Biodiversidad, Facultad de Química y Biología, Universidad de Santiago de Chile (USACH), Santiago 9170022, Chile; cristian.ray.b@gmail.com (C.R.); mathias.alfaro@usach.cl (M.A.); 2 Centro de Investigaciones Arquitectónicas, Urbanísticas y del Paisaje (CEAUP), Universidad Central de Chile, Santiago 8370178, Chile; javier.figueroa@ucentral.cl; 3 Corporación Jardín Botánico Chagual, Comodoro Arturo Merino Benitez N°3020, Vitacura, Santiago 7630213, Chile; florrego@uc.cl; 4 Departamento de Gestión Agraria, Universidad de Santiago de Chile (USACH), Santiago 9170022, Chile; pablo.vergara@usach.cl

**Keywords:** exotic plant species, native plant species, parks, sidewalks, socioeconomic level, vacant lots

## Abstract

Cities host a heterogeneous composition of native and exotic plants, yet the spatial distribution of plant richness and its drivers remain poorly understood. We evaluated the influence of the center-edge gradient, along the environmental gradient from the historic city center to the urban edge, and habitat type, reflecting local conditions, on plant richness in Santiago, Chile. Sidewalks, parks, and vacant lots (*n* = 234 per habitat type) were randomly sampled across varying distances from the historic center and urban edge, recording neighborhood socioeconomic level and municipality. Four richness metrics were analyzed using generalized linear mixed models (GLMMs): total richness, richness by origin (native or exotic), and richness by life form (trees, shrubs, or herbs), considering habitat type, socioeconomic level, and distances as fixed effects and municipality as a random effect. We recorded 699 species (13% native and 87% exotic; 23% trees, 20% shrubs, and 56% herbs). Distances to the city center and urban edge had no significant effect, whereas habitat type emerged as the primary determinant: sidewalks exhibited higher total, native, and exotic richness with more trees and shrubs, whereas vacant lots were dominated by herbs. These patterns indicate that floristic richness is distributed in a mosaic, independent of urban gradients. Given the importance of Santiago’s Mediterranean region as a biodiversity hotspot, the low representation of native species is concerning. Increasing their presence and associated ecosystem services requires tailored interventions for each habitat type.

## 1. Introduction

Urbanization is one of the main drivers of biodiversity change at a global scale [1,2,3]. Cities modify habitats for native species, restricting their presence to those capable of tolerating or exploiting novel urban conditions [4,5,6]. On the other hand, urban areas also facilitate the introduction of exotic species (i.e., species originating from other regions), facilitating their establishment and persistence [7,8]. Consequently, cities host variable combinations of species with diverse phylogenetic and biogeographic origins [9].

Vascular plants are among the most ubiquitous and conspicuous taxonomic components of urban biotas [10,11]. Ecologically, they provide habitat and resources for animals, fungi, and microorganisms [12,13,14] and deliver key ecosystem services relevant to human well-being [15,16,17]. These functions have driven growing interest in describing urban floristic diversity and understanding the determinants of richness, abundance, and distribution [18,19,20]. However, most of the current evidence comes from cities in the Northern Hemisphere [21,22], creating a geographic bias that limits our understanding of urbanization effects on floristic diversity, the integration of cities into plant conservation efforts [23,24,25], and their transit toward more sustainable socioecosystems [16,26,27].

From a spatial perspective, urban floristic richness can be organized in two ways. First, it may vary in a (quasi- or pseudo-)monotonic fashion along the center-edge gradient, consistent with environmental gradients that typically change in the same direction [28,29,30,31,32], due, for example, to soil sealing, concentration of grey infrastructure, urban heat island intensification, and increased anthropogenic disturbances [33]. Second, richness may depend on the heterogeneity of available habitat types, which, without necessarily following the center-edge trend, provide diverse conditions of vegetation management and control [34,35,36]. In this case, richness may exhibit a “mosaic” pattern *sensu* [34] associated, for example, with different habitat types (e.g., parks, gardens, and vacant lots), whose characteristics typically reflect local management, income level, aesthetic preferences, among other factors [10,28,34]. Quantifying the relative contribution of center-edge gradients and local habitat heterogeneity within a city is a key step toward understanding the distribution of urban floristic richness, assuming that these effects are not mutually exclusive but may occur simultaneously.

Santiago of Chile, the sixth most populous city in South America [37], is located in a global biodiversity hotspot [38] characterized by a high level of plant endemism (~50%; [39]). Nevertheless, regional floristic richness is scarcely represented within the city, as <1% of species present in the administrative region (approximately 1400 species) occur in the urban area [40]. Recent records indicate that around 13% of species in Santiago are native (76 species, [41]), one of the lowest proportions reported for urban areas globally [42] (and references therein). Additionally, previous studies show that tree species diversity is associated with socioeconomic level [43,44,45,46,47,48,49], with richness increasing from lower- to higher-income municipalities. Cartographic analyses have also identified local hotspots of high native and exotic plant richness [50], suggesting a key role of habitat types in shaping urban diversity. However, knowledge gaps remain regarding how floristic richness is distributed across the city, particularly along the center-edge gradient, as well as the relative importance of different habitat types. Addressing these questions is critical for understanding urban flora distribution and for guiding the planning of green infrastructure and the conservation of native species in cities.

In this study, we analyze Santiago’s urban flora in its entirety, including both spontaneously established and deliberately planted vascular plants. Specifically, we analyzed spatial patterns of floristic richness in Santiago, assessing how native and exotic species, as well as their life forms, respond to the center-edge gradient and local habitat heterogeneity. Based on evidence from other cities, we expected that total richness and its components by origin and life form would respond to the center-edge gradient, increasing with distance from the historic city center. We also anticipated that habitat types would differ in richness, with managed habitats (e.g., sidewalks and parks) exhibiting higher species diversity than unmanaged habitats (e.g., vacant lots). Finally, we hypothesized that the effects of socioeconomic level, previously documented for tree species, could also extend to other life forms. Overall, the results of this study provide valuable insights into the primary factors influencing urban floristic richness, with applications for conservation and green infrastructure management, emphasizing the promotion of native flora.

## 2. Methods

### 2.1. City of Santiago

**Figure 1:**
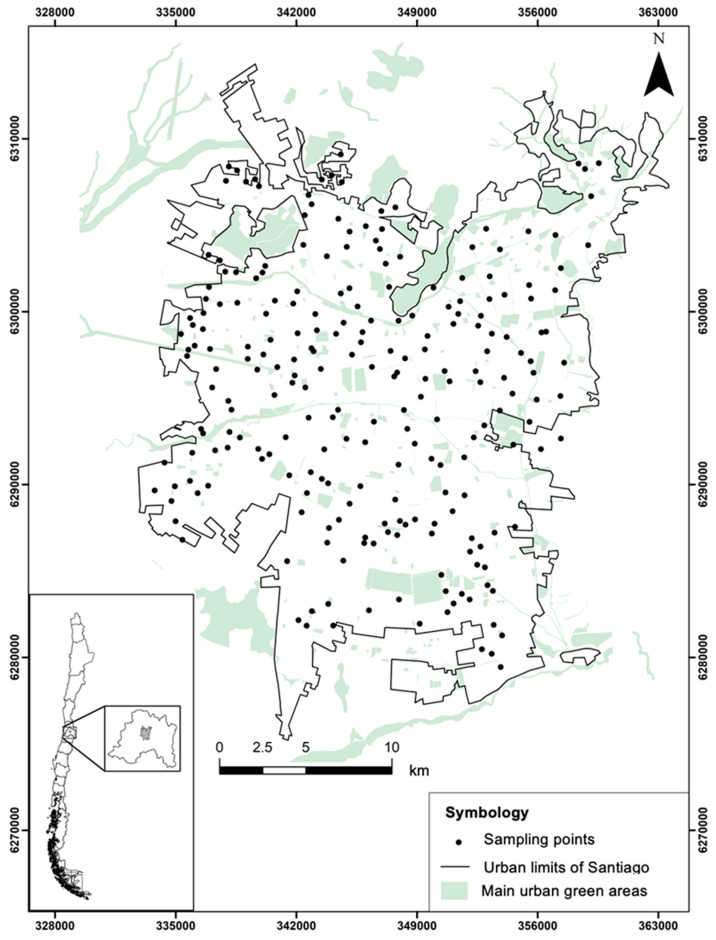
Geographic distribution of the 234 sampled points within the urban area of Santiago.

Santiago (Figure 1) is located in central Chile, within the administrative unit known as the Metropolitan Region (RM). The city covers an area of 837.9 km^2^ [51] (Instituto Nacional de Estadísticas de Chile, 2017) and lies at a mean elevation of 520 m a.s.l. [52]. Its climate is Mediterranean, with hot, dry summers and cold, rainy winters, registering an annual mean temperature of 15.1 °C and an average annual precipitation of 261 mm (data from 2000–2023; [53]).

The city was founded in 1541 by Spanish colonists on a pre-existing Inca settlement [54], located in the Mapocho River valley, which constitutes its main watercourse. The original native vegetation likely consisted of sclerophyllous Mediterranean forest formations [40], characterized by trees such as peumo (*Cryptocarya alba*), litre (*Lithraea caustica*), boldo (*Peumus boldus*), and quillay (*Quillaja saponaria*), adapted to strongly seasonal precipitation primarily occurring in winter and high summer temperatures [55]. Currently, the city is surrounded by a matrix of rural lands undergoing transformation, where agricultural, industrial, and low-density residential uses coexist [46]. According to the most recent national census [51], the city has 7,112,808 inhabitants, representing approximately 40% of the national population. Thus, Santiago is the most populous city in Chile and the sixth largest metropolitan area in South America [37].

### 2.2. Floristic Sampling

To characterize the spatial pattern of floristic richness, sampling was conducted between 2014 and 2025 during spring and summer, coinciding with the period of maximum vegetation growth [41]. A total of 234 points were randomly distributed across Santiago (Figure 1) using QGIS 3.x [56] and the Random Points Inside Polygons (with Minimum Distance) plugin over a polygon delineating the urban extent. Each point was established with a minimum separation of 300 m from other points. From these reference points, the three nearest habitat types were located: sidewalks (234), parks (234), and vacant lots (234), which were surveyed exhaustively (see Supplementary Material Table S1). These counts reflect the current tally, as the survey is ongoing; the dataset reported here covers samples collected through 2025. Sidewalks were defined as pedestrian strips on both sides of the street. Parks were publicly accessible green areas maintained by municipalities for recreational purposes. Vacant lots were open-access areas without active management or in a state of abandonment. It is important to note that we did not implement a standardized plot-size sampling scheme due to the marked differences in geometry and available surface across the city; moreover, using a single plot size would have located a substantial fraction of potential sites on private property, outside public space and, therefore, beyond the scope of our study. Nevertheless, to control for area effects, we modeled species richness using GLMMs and included log(area) as an offset, so that cross-habitat comparisons are equivalent to standardization per unit area (see Statistical Analysis).

For all three habitat types, complete surveys were conducted, considering both sidewalks in the case of streets. Since site sizes varied, the effectively sampled area (m^2^) was recorded for each site, ranging from 13 to 506,238 m^2^ with a median of 1164 m^2^. Specifically, sidewalks ranged from 161 to 4076 m^2^ (both sidewalks combined), parks from 226 to 24,527 m^2^, and vacant lots from 13 to 506,238 m^2^.

Each site was georeferenced, and Euclidean distances (km) to the historic city center (Plaza de Armas), to the nearest urban edge (defined as the limit of urbanized area), and elevation (m a.s.l.) were estimated using Google Earth Pro [57]. To characterize the socioeconomic status of each site, we used the Santiago Economic Perceptions Study [58], which classifies neighborhoods into five segments: ABC1 (high class), C2 (typical middle class), C3 (lower middle class), D (vulnerable middle class), and E (low class). To ensure balanced sample sizes, categories D and E were combined (DE).

The distribution of the number of habitat types sampled by socioeconomic level is shown in Supplementary Material Table S1.

### 2.3. Flora

At each site, we recorded both spontaneously established and intentionally planted vascular plant species (herbs, shrubs, and trees) identified in the field and entered into a database. Specimens with uncertain identification were collected for subsequent taxonomic determination in the laboratory. Nomenclature followed World Flora Online [59], and synonyms were verified using Plants of the World Online [60]. Each species was classified as either native or exotic according to its biogeographic origin, following Santilli et al. [61] and references therein. Species whose natural distribution includes central Chile, and which are part of the local vegetation formations surrounding Santiago were considered native [40]. A small number of species native to other regions of Chile (*n* = 4), also known as extralimital species *sensu* [62], were included in the native group. Exotic species included all those introduced from other regions of the world (intentionally or accidentally), including ornamental, naturalized, or spontaneous species [41,61]. Finally, 16 taxa were excluded from analyses because they were only identified to genus level.

### 2.4. Statistical Analysis

To evaluate the effects of the center-edge gradient and habitat heterogeneity on floristic richness, site-level total richness, native richness, exotic richness, and richness by life form (trees, shrubs, herbs) were modeled using GLMMs with a log link, assuming a negative binomial (NB2) distribution for each response variable. A log(area) offset was included to control for differences in sampled area, and a random intercept for municipality (1|municipality) was included to capture unobserved variation and potential spatial dependence.

Fixed effects included habitat type (sidewalk, park, vacant lot), socioeconomic level (ABC1, C2, C3, DE), and standardized distances to the historic center and urban edge (mean = 0, SD = 1). Interactions among habitat, socioeconomic level, and distances were included but were mostly non-significant; hence we focused on additive effects to be consistent with our hypotheses regarding direct effects. Collinearity among continuous covariates was low (VIF < 3). Potential non-linear effects of distances (quadratic terms, exponential transformations, and natural splines with df = 3) were explored, but none improved model fit relative to the linear model (ΔAIC = +1.25; LRT *p* = 0.150), so the most parsimonious specification was adopted.

Zero-truncated NB2 families were used for responses without zeros (total richness, exotics, herbs) and standard NB2 for natives, trees, and shrubs. DHARMa diagnostics (dispersion, uniformity, outliers, and zero inflation) guided model extensions: zero-inflation components were included only when indicated by residuals and if model fit improved by at least 2 AIC units (ΔAIC ≤ −2). Effects are reported as rate ratios (RR = e^β^) with 95% confidence intervals and *p*-values. Post hoc comparisons among habitat and socioeconomic levels were conducted using emmeans (Tukey-adjusted) on the response scale. All analyses were performed in R Core Team [63] using the packages glmmTMB [64], DHARMa [65], emmeans [66], broom.mixed [67], and dplyr [68].

## 3. Results

Across the 702 sampled sites, we recorded 699 vascular plant species, of which 13% were native and 87% exotic (Table 1). Of these species 161 were trees (23%), 146 shrubs (21%), and 392 herbs (56%) (Table 1); trees were present in 92% of sites, shrubs in 71.9%, and herbs in 100% of sites. The distribution of taxonomic richness across habitat types (number species per habitat or α-diversity) showed that representation by origin did not differ from the overall city pattern (γ-diversity or entire pool species), but representation by life form did (Table 1). Indeed, both sidewalks and parks had a higher representation of trees and shrubs and lower herb richness (Table 1), whereas herbs were concentrated in vacant lots, with lower richness on sidewalks and in parks (Table 1). Species richness per habitat ranged from 2 to 114 (median = 28); native richness ranged from 0 to 17 (median = 2) and exotic richness from 1 to 102 (median = 26).

**Figure 2:**
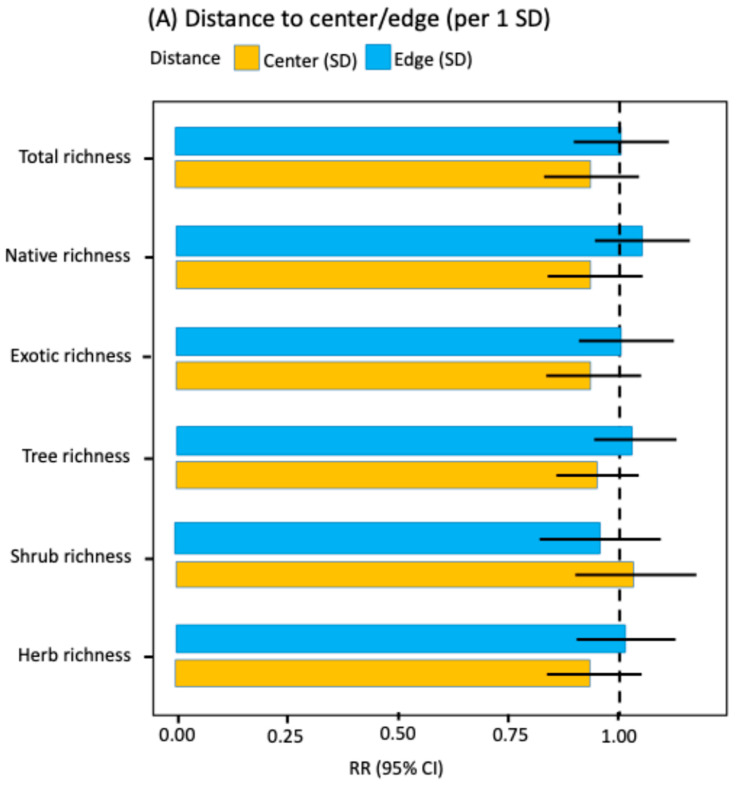

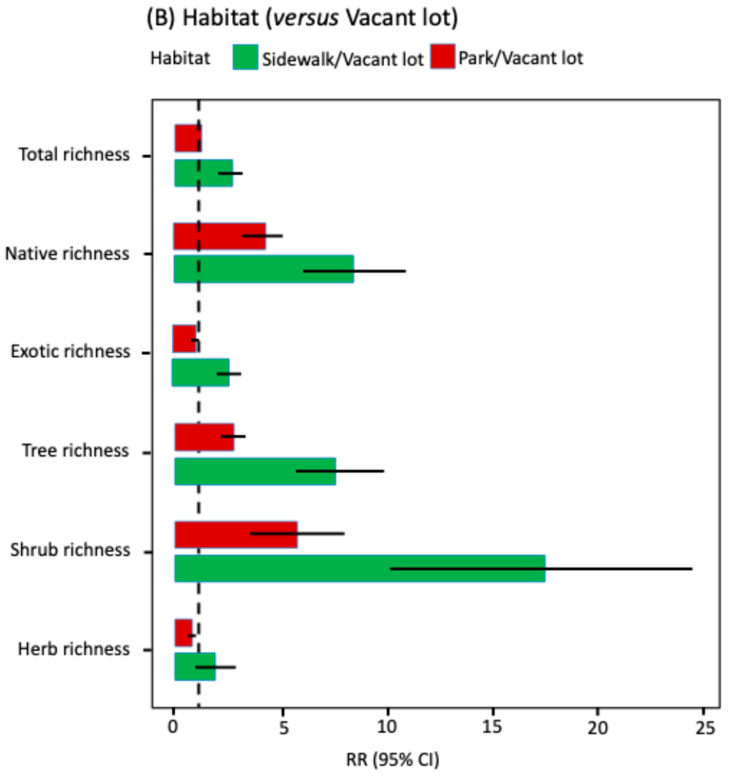

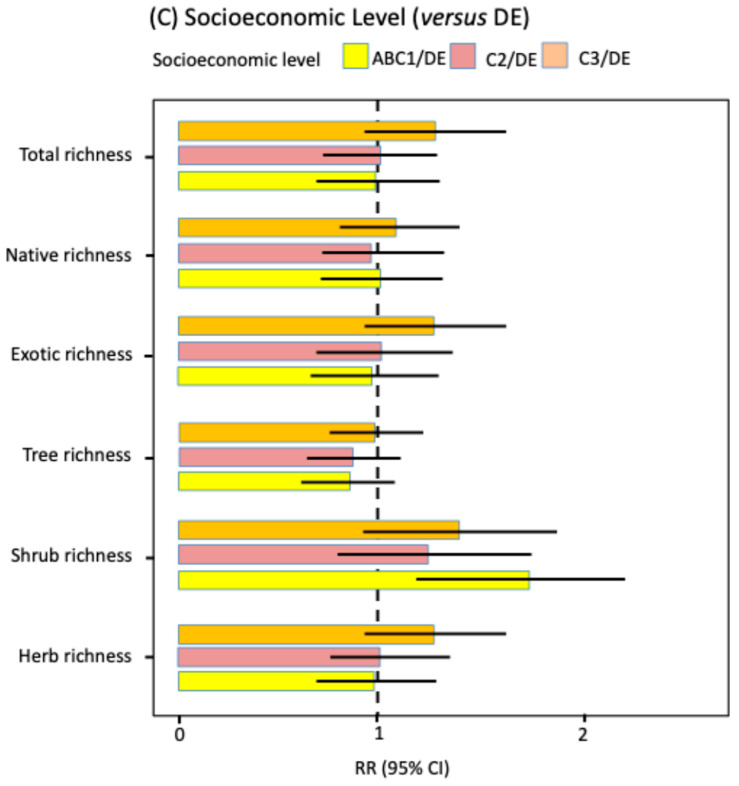
Rate ratios (RR) with 95% confidence intervals for key predictors of species richness (GLMM NB2, log link, log [area] offset). (**A**) Spatial gradients: effects per one-standard deviation (SD) of distance to the city center (orange) and distance to the urban edge (blue); (**B**) Habitat (reference: vacant lot): sidewalk (green) and park (red); (**C**) Socioeconomic level (reference: DE): ABC1 (orange), C2 (purple), and C3 (yellow). Continuous covariates were z-standardized (mean 0, SD 1). Points show RR; bars show 95% CI. The dashed line indicates RR = 1 (no effect relative to the reference).

GLMM analyses indicated that neither distance to the historic city center nor distance to the urban edge had significant effects on total floristic richness, richness by origin, or richness by life form (Figure 2A; Supplementary Material Table S2). In all cases, an increase of one-standard deviation in distance from the city center or the urban edge had no significant effect on floristic richness (Figure 2A; Supplementary Material Table S2). Thus, the radial center-edge axis did not reveal any systematic patterns of richness variation at the city-wide scale.

In contrast, habitat type emerged as the main determinant (Figure 2B; Supplementary Material Table S2). When vacant lots were used as a reference, sidewalks exhibited higher expected species richness per site for total, native, and exotic richness, as well as for trees and shrubs. Parks surpassed vacant lots in native species and woody species (trees and shrubs), whereas vacant lots showed greater representation of herbs than parks. These patterns were confirmed by Tukey post hoc contrasts (Supplementary Material Table S3).

The effects of socioeconomic segment were weak and inconsistent across response variables (Figure 2C; Supplementary Material Table S2). After adjustment for multiple comparisons using the Tukey test, only one significant difference was observed: higher shrub richness in ABC1 segments compared to DE segments (RR = 1.75, *p*-adj = 0.004; Supplementary Material Table S3). GLMM contrasts without adjustment showed some trends (lower shrub richness in C2 and DE versus ABC1; RR ≈ 0.68 and 0.57; and higher herb and exotic richness in C3 versus ABC1; RR ≈ 1.37 and 1.32; Supplementary Material Table S3), but these effects were not significant after multiplicity correction. Consequently, except for the difference between ABC1 and DE for shrubs, no other significant differences were detected among the richness indicators.

Finally, of the interactions evaluated, only a few were significant: vacant lot × socioeconomic segments in C2 and C3 (RR = 1.94 and 2.34, respectively; Supplementary Material Table S3), indicating that richness in vacant lots is higher in these socioeconomic segments than suggested by the main habitat effects. Additionally, the interaction between vacant lot and distance to the center (RR = 0.793; Supplementary Material Table S3) indicated a slight decrease in richness in vacant lots with increasing distance from the city center. All other interactions were non-significant, confirming that the main patterns are primarily explained by the direct effects of habitat, socioeconomic segments, and distances. Similar results were observed for the other richness metrics (native, exotic, and by life form), with most interactions not reaching significance.

## 4. Discussion

Several studies have shown that floristic richness and abundance can respond to the urban center-periphery gradient [3,11,31]. This pattern has been attributed to environmental variations along the radial distance from the historic center to the urban edge [4,33], including factors such as temperature, humidity, grey or green infrastructure, and land-use history [9,43]. In Santiago, however, none of the richness indicators evaluated showed significant as-sociations with distance to either the historic center or the urban edge. The result holds under our broadly inclusive treatment of the city’s urban flora, encompassing both planted and spontaneously established species, and remained robust when we fitted generalized linear mixed models (GLMMs) with nonlinear terms (see Methods). Therefore, previously documented urban environmental gradients in the city-such as temperature, particulate matter, grey infrastructure, or population density, among others [69,70], do not translate into site-level responses in plant richness.

In contrast, habitat type (sidewalks, parks, and vacant lots) emerged as the main predictor of floristic richness in Santiago, consistent with studies in cities in China [71,72,73], Central Europe [34,74], and the United States [75]. Accordingly, the distribution of plant diversity follows a mosaic pattern *sensu* [10], consistent with the existence of multiple local centers of high diversity within the city, as previously described by Castro et al. [50].

It is particularly noteworthy that sidewalks systematically concentrate the highest richness per unit area, approximately 2.5 times higher than parks and vacant lots. This pattern can be explained, firstly, by municipal management, responsible for public space landscaping, which predominantly incorporates trees along sidewalks. Secondly, resident interventions add mainly ornamental shrubs and herbs, and in some cases create “sidewalk gardens”, enhancing local diversity [76]. Thirdly, sidewalks act as dispersal corridors for various ruderal species [77], which can establish and persist due to the microenvironmental heterogeneity of this habitat type, including cracks, pavement edges, and small soil patches.

Parks tended to host higher richness of native species and woody plants (trees and shrubs) than vacant lots, while exotic species richness did not differ significantly between these habitats. Herbs were more abundant in vacant lots. This contrast, by origin and life form, reflects different management regimes. Parks, with standardized designs and intensive management (irrigation, lawns, pruning, and weed control), favor municipally planted species, often biased towards a broad range of exotics and with limited representation of native taxa in trees [49,78]. In contrast, vacant lots, subject to minimal or no management, sustain communities dominated by drought-tolerant ruderal herbs and species tolerant to local disturbances, such as soil compaction, removal, or fires [77,79,80].

Interactions between habitat, socioeconomic level, and spatial location also revealed relevant patterns in vacant lots. Although these sites generally show lower richness than sidewalks and parks, observed richness in C2-C3 neighborhoods exceeded expectations based on the main habitat effect. This may reflect higher recent turnover or abandonment in these strata, favoring early successional states where ornamental remnants coexist with colonizing ruderal species. Additionally, the interaction with distance to the city center suggests that vacant lots closer to the urban core show slightly higher richness, likely due to greater propagule pressure and connectivity with other green areas. Overall, most interactions were not statistically significant, indicating that observed patterns are primarily driven by main effects. However, these results highlight that in certain combinations of factors, floristic richness can deviate from dominant patterns, emphasizing the localized importance of interactions among habitat, socioeconomic level, and spatial location.

Unlike other studies e.g., [81,82,83,84], including work in Santiago [44,45,46] reporting the “luxury effect” [85], we found no evidence that socioeconomic level influenced total floristic richness or its components by origin (native, exotic) or life form (trees, shrubs, herbs). The sole exception was shrub richness, whose median was approximately 50% higher in ABC1 neighborhoods (high socioeconomic level) compared to DE neighborhoods (low socioeconomic level); other comparisons among socioeconomic levels were not significant. While in Santiago, socioeconomic level correlates with greater vegetation cover at the neighborhood scale [79], this does not necessarily translate into increased richness at the scale of the habitats considered in this study. Similarly, Guevara et al. [49] found no significant differences in total tree richness or abundance across socioeconomic levels in urban green areas, though they observed differences by species origin: exotic species showed uniform richness and abundance, whereas native species were richer and more abundant in high- versus low-income areas.

Discrepancies in detecting the “luxury effect” in Santiago may be explained by at least three factors. First, the high heterogeneity among sites and habitat types-defined here by composition, structure, disturbance history, size, and management-broadens within-group variation (within each socioeconomic level), diluting the effect. This does not preclude its presence in specific habitat types not considered in this study, such as certain green areas [49]. Second, methodological differences relative to previous studies may contribute. While earlier work assigned socioeconomic level at the municipal scale and used fixed-size plots without controlling for covariates, our approach incorporated finer-grain sampling at the neighborhood level, including sidewalks, parks, and vacant lots, accounting for effective area and using GLMMs with random effects for municipality and site. Third, compensatory processes occur along the socioeconomic gradient. In lower strata (C3, DE), lower investment by residents and municipalities favors the persistence of ruderal and spontaneous species. In higher strata (ABC1, C2), intensive management and introduction of ornamentals tend to exclude these ruderal species, although ornamental presence increases. Collectively, heterogeneity, methodological resolution, and compensatory processes may explain why we did not detect significant socioeconomic effects on floristic richness.

In Santiago, urban flora is strongly dominated by exotic species (87%), with natives representing only 13%, one of the most pronounced disparities reported in urban systems [42,61,62]. Novel to our study, this ratio remains stable across habitat types analyzed (sidewalks, parks, vacant lots), with natives comprising 12–13% and exotics 87–88%. This consistent urban-scale pattern provides a comprehensive view of the city’s floristic structure. Exotic dominance is likely underestimated here, as individual abundance was not considered, which would further skew representation toward exotics. Additionally, four extralimital native species (araucaria, *Araucaria araucana*; olivillo, *Aextoxicon punctatum*; pilo, *Sophora cassioides*; and verónica, *Hebe salicifolia*), endemic to Chile but outside Santiago’s biogeographic region, were classified as native. Overall, these results reflect a marked imbalance in urban native flora representation, with important implications for green space conservation and planning.

What explains the low representation of native species in Santiago? We propose the combined action of at least two major urban filters (*sensu* [11]). First, a sociocultural filter, based on ornamental norms established since the mid-19th century that emulated European models and historically favored the introduction and planting of exotic species [80]. This bias is reinforced by the limited availability of native flora in nurseries and cultural preferences for introduced species [78]. Although native plantings—mainly trees such as peumo (*Cryptocarya alba*), boldo (*Peumus boldus*), espino (*Vachellia caven*), pimiento (*Schinus molle*), and quillay (*Quillaja saponaria*)—have been promoted in the last two decades, these efforts remain localized and limited. Second, an ecological filter, stemming from historical landscape transformation in central Chile since European colonization (1541), characterized by progressive replacement of native vegetation with agro-silvicultural matrices over approximately 480 years [86,87]. The current urban habitat presents strongly modified environmental conditions that hinder the establishment of native sclerophyllous forest species, whose functional requirements for soil, water, light, and biotic interactions (pollination, dispersal, nutrient cycling, among others) make survival in the city improbable [88]. Together, these sociocultural and ecological filters explain the marked scarcity of native urban flora and highlight the need for planning and restoration strategies that consider both environmental limitations and historical cultural dynamics.

Life forms are unevenly represented across habitats in Santiago. Herbs dominate, accounting for 56% of recorded urban species, a proportion largely overlooked in Santiago’s urban literature, which has focused mainly on trees [44,45,46,49]. Most herbs (57%) occur in vacant lots, reflecting their largely spontaneous or ruderal nature [77], while smaller fractions occur on sidewalks and in parks (20% and 11%, respectively), where ornamental species predominate. Woody species (trees and shrubs) constitute 23% and 21% of city-wide floristic diversity, concentrated mainly on sidewalks and in parks (74% and 88% for trees; 81% and 69% for shrubs), reflecting the predominance of ornamental species in habitats designed for urban beautification.

This compositional pattern by life form is especially relevant given climate change projections for central Chile [89,90], which predict sustained precipitation decreases and greater constraints on urban water availability [91]. Under these conditions, several native species exhibit higher drought tolerance, whereas most exotic ornamentals require frequent irrigation due to their origin in wetter regions [61]. This functional difference highlights the dependence of exotics on intensive irrigation and the efficiency of natives under water-limited conditions [88]. Furthermore, Arcos-LeBert et al. [15] demonstrated that ecosystem services provided by native trees surpass those exotics under water-limited scenarios, reinforcing the need to integrate natives into the urban landscape to increase ecological resilience and optimize water use.

The results of this study have important implications for urban flora conservation and management in Santiago. Although native species represent approximately 13% of the city’s flora, this proportion accounts for less than 1% of the regional floristic diversity of central Chile [40], a globally significant biodiversity hotspot [38]. Despite this low relative representation, the city holds strategic potential as a refuge and conservation area for native species [11,92]; thus, urban planning can incorporate regional preservation objectives. Promoting native plants in sidewalks, parks, and vacant lots would not only increase local representation but also connect the city to surrounding biodiversity conservation efforts.

Furthermore, identifying habitat type as the main determinant of floristic richness indicates that site-scale design and management can amplify ecological efficiency, maximizing both native species representation and the ecosystem services they provide. Incorporating ecological criteria into spatial planning instruments, promoting availability of native flora in urban nurseries, and tailoring management to the characteristics of each habitat could transform the city into a more sustainable, functional space committed to regional biodiversity conservation.

## 5. Conclusions

In Santiago, 699 urban plant species were recorded, dominated by exotics (87%) with only 13% natives, representing less than 1% of the regional diversity of central Chile. Richness does not follow a radial center-edge gradient but is distributed in local mosaics, with habitat type (sidewalks, parks, and vacant lots) as the main determinant of diversity. These patterns reflect the combined influence of historical, ecological, and sociocultural factors that have favored exotic dominance and modulated the presence of native taxa. Under projected global change and water stress scenarios for central Chile, these results highlight the importance of promoting native species adapted to water scarcity and valuing urban habitat heterogeneity. Incorporating these considerations into the design and management of green infrastructure can enhance ecological resilience, optimize ecosystem services, and contribute to regional biodiversity conservation in urban contexts.

## Figures and Tables

**Table 1:** Plant species richness across habitat types in Santiago, stratified by biogeographic origin and life form. The Total column reports the species pool with the same stratification. Chi-square tests of homogeneity (*χ*^2^, df, *p*) evaluating whether richness is uniformly distributed across habitat types are reported in the table footnotes (notes a,b).

	Sidewalks	Parks	Vacant Lots	Total (%)
**Biogeographic origin ^(*a*)^**				
Native	66	75	47	93 (13%)
Exotic	483	468	302	606 (87%)
Total	549	543	349	699 (100%)
				
**Life form ^(*b*)^**				
Tree	120	143	81	161 (23%)
Shrub	119	102	45	146 (21%)
Herb	81	45	223	392 (56%)
Total	320	290	349	699 (100%)

(*a*) *χ*^2^ = 0.843; df = 2; *p* = 0.656; (*b*) *χ*^2^ = 192.2; df = 4; *p* < 0.001.

## Data Availability

The original contributions presented in this study are included in the article/Supplementary Material. Further inquiries can be directed to the corresponding author.

## Supplementary Materials

The following supporting information can be downloaded at: https://www.mdpi.com/article/10.3390/plants14223433/s1, S1. Counts of sampled sites by habitat type and socioeconomic level (*n* = 702); S2. Generalized linear mixed model (GLMM, negative binomial NB2, log link) predicting species richness per site, with log-transformed area included as an offset. Rate ratios (RR = e^β^) are pre-sented with 95% confidence intervals (CI) and associated *p*-values. Reference categories were sidewalk for habitat and ABC1 for socioeconomic level. Continuous predictors are standardized (1 SD). SEL: Socioeconomic Level; S3. Tukey post hoc comparisons (emmeans) among habitat levels on the response scale. Rate ratios (RR = e^β^), 95% confidence intervals (CI), and adjusted *p*-values are reported. RR > 1 indicates that the first habitat in the contrast has higher species richness than the second. Models are based on those in Table 1, with log(area) included as an offset, a random intercept for municipality, and standardized covariates.
