# Supplementary material for: Effects of the Center-Edge Gradient and Habitat Type on the Spatial Distribution of Plant Species Richness in Santiago, Chile
Source: Plants (Basel). 2025 Nov 10;14(22):3433. doi: 10.3390/plants14223433 (PMC12656292; doi:10.3390/plants14223433)
Supplement: Supplementary file 1 [file plants-14-03433-s001.zip › plants-3962117-supplementary.pdf]

Table S1. Counts of sampled sites by habitat type and socioeconomic level (n = 702).

	Socieconomic level				
	ABC1	C2	C3	DE	Total
Sidewalk	44	54	68	68	234
Park	44	54	68	68	234
Vacant lot	44	54	68	68	234
Total	132	162	204	204	702

Table S2. Generalized linear mixed model (GLMM, negative binomial NB2, log link) predicting species richness per site, with log-transformed area included as an offset. Rate ratios (RR = e^{β}) are presented with 95% confidence intervals (CI) and associated p-values. Reference categories were sidewalk for habitat and ABC1 for socioeconomic level. Continuous predictors are standardized (1 SD). SEL: Socioeconomic Level.

**Response/Term**	**RR**	**CI95% infe-rior**	**IC95% su-perior**	***z***	***p***
**Total richness**					
Habitat: Park (*versus* Sidewalk)	0.373	0.309	0.451	-10.25	<0.001
Habitat: Vacant lot (*versus* Sidewalk)	0.395	0.323	0.484	-9.01	<0.001
SEL: C2 (*versus* ABC1)	1.036	0.791	1.358	0.26	0.797
SEL: C3 (*versus* ABC1)	1.302	1	1.696	1.96	0.05
SEL: DE (*versus* ABC1)	1.026	0.79	1.331	0.19	0.849
Distance to urban centre (per 1 SD)	0.954	0.856	1.063	-0.86	0.392
Distance to urban edge (per 1 SD)	1.024	0.922	1.137	0.44	0.659
**Native richness**					
Habitat: Park (*versus* Sidewalk)	0.338	0.282	0.404	-11.87	<0.001

Habitat: Vacant lot (*versus* Sidewalk)	0.133	0.106	0.168	-16.97	<0.001
SEL: C2 (*versus* ABC1)	0.978	0.743	1.288	-0.16	0.875
SEL: C3 (*versus* ABC1)	1.067	0.811	1.402	0.46	0.643
SEL: DE (*versus* ABC1)	1.014	0.782	1.314	0.1	0.917
Distance to urban centre (per 1 SD)	0.953	0.857	1.06	-0.88	0.378
Distance to urban edge (per 1 SD)	1.07	0.967	1.184	1.3	0.193
**Exotic richness**					
Habitat: Park (*versus* Sidewalk)	0.372	0.312	0.444	-11.01	<0.001
Habitat: Vacant lot (*versus* Sidewalk)	0.407	0.336	0.491	-9.3	<0.001
SEL: C2 (*versus* ABC1)	1.055	0.816	1.364	0.41	0.684
SEL: C3 (*versus* ABC1)	1.323	1.029	1.7	2.18	0.029
SEL: DE (*versus* ABC1)	1.05	0.819	1.345	0.38	0.702
Distance to urban centre (per 1 SD)	0.957	0.861	1.065	-0.81	0.421
Distance to urban edge (per 1 SD)	1.024	0.924	1.134	0.45	0.653
**Tree richness**					
Habitat: Park (*versus* Sidewalk)	0.495	0.421	0.581	-8.56	<0.001
Habitat: Vacant lot (*versus* Sidewalk)	0.123	0.1	0.152	-19.64	<0.001
SEL: C2 (*versus* ABC1)	1.012	0.789	1.298	0.1	0.923
SEL: C3 (*versus* ABC1)	1.154	0.904	1.474	1.15	0.251
SEL: DE (*versus* ABC1)	1.154	0.911	1.463	1.19	0.235
Distance to urban centre (per 1 SD)	0.979	0.892	1.074	-0.45	0.652
Distance to urban edge (per 1 SD)	1.049	0.959	1.147	1.04	0.298
**Shrub richness**					
Habitat: Park (*versus* Sidewalk)	0.324	0.261	0.403	-10.2	<0.001
Habitat: Vacant lot (*versus* Sidewalk)	0.057	0.042	0.077	-18.93	<0.001
SEL: C2 (*versus* ABC1)	0.679	0.483	0.954	-2.23	0.025

SEL: C3 (*versus* ABC1)	0.781	0.56	1.089	-1.46	0.145
SEL: DE (*versus* ABC1)	0.571	0.411	0.792	-3.36	<0.001
Distance to urban centre (per 1 SD)	1.035	0.902	1.188	0.49	0.622
Distance to urban edge (per 1 SD)	0.977	0.853	1.119	-0.34	0.735
**Herb richness**					
Habitat: Park (*versus* Sidewalk)	0.339	0.282	0.406	-11.68	<0.001
Habitat: Vacant lot (*versus* Sidewalk)	0.482	0.397	0.586	-7.33	<0.001
SEL: C2 (*versus* ABC1)	1.101	0.843	1.439	0.7	0.481
SEL: C3 (*versus* ABC1)	1.37	1.056	1.778	2.37	0.018
SEL: DE (*versus* ABC1)	1.071	0.828	1.386	0.52	0.6
Distance to urban centre (per 1 SD)	0.948	0.848	1.061	-0.93	0.352
Distance to urban edge (per 1 SD)	1.029	0.923	1.146	0.51	0.609

Table S3. Tukey post hoc comparisons (emmeans) among habitat levels on the response scale. Rate ratios (RR = e^{β}), 95% confidence intervals (CI), and adjusted p-values are reported. RR > 1 indicates that the first habitat in the contrast has higher species richness than the second. Models are based on those in Table 1, with log(area) included as an offset, a random intercept for municipality, and standardized covariates.

Factor/Response	Contrast	Rate Ratio (RR)	*p*-adj
**Habitat**			
Total richness	Sidewalk/Park	2.678	<0.001
Herb richness	Sidewalk/Vacant lot	2.074	<0.001
Herb richness	Sidewalk/Park	2.954	<0.001

Shrub richness	Park/Vacant lot	5.692	<0.001
Shrub richness	Sidewalk/Vacant lot	17.561	<0.001
Shrub richness	Sidewalk/Park	3.085	<0.001
Tree richness	Park/Vacant lot	4.009	<0.001
Tree richness	Sidewalk/Vacant lot	8.102	<0.001
Tree richness	Sidewalk/Park	2.021	<0.001
Herb richness	Park/Vacant lot	0.702	0.001
Exotic richness	Sidewalk/Vacant lot	2.46	<0.001
Exotic richness	Sidewalk/Park	2.687	<0.001
Native richness	Park/Vacant lot	2.531	<0.001
Native richness	Sidewalk/Vacant lot	7.494	<0.001
Native richness	Sidewalk/Park	2.961	<0.001
Total richness	Park/Vacant lot	0.944	0.844
Total richness	Sidewalk/Vacant lot	2.529	<0.001
Exotic richness	Park/Vacant lot	0.915	0.633
**Socioeconomic level**			
Tree richness	ABC1/C3	0.867	0.66
Tree richness	ABC1/DE	0.866	0.635
Tree richness	C2/C3	0.877	0.627
Tree richness	C2/DE	0.877	0.627
Tree richness	C3/DE	1	1
Shrub richness	ABC1/C2	1.473	0.115
Shrub richness	ABC1/C3	1.28	0.464
Shrub richness	C3/DE	1.369	0.112
Shrub richness	C2/C3	0.869	0.801
Shrub richness	C2/DE	1.19	0.693
Herb richness	ABC1/C2	0.908	0.895
Herb richness	ABC1/C3	0.73	0.084

Herb richness	ABC1/DE	0.933	0.953
Herb richness	C2/C3	0.804	0.25
Tree richness	ABC1/C2	0.988	1
Shrub richness	ABC1/DE	1.753	0.004
Exotic richness	C3/DE	1.26	0.133
Native richness	ABC1/DE	0.986	1
Exotic richness	C2/C3	0.797	0.195
Total richness	ABC1/C2	0.965	0.994
Total richness	ABC1/C3	0.768	0.205
Total richness	ABC1/DE	0.975	0.998
Total richness	C2/C3	0.796	0.231
Total richness	C2/DE	1.01	1
Total richness	C3/DE	1.269	0.15
Native richness	ABC1/C2	1.022	0.999
Native richness	ABC1/C3	0.937	0.967
Herb richness	C2/DE	1.028	0.996
Native richness	C2/C3	0.917	0.896
Native richness	C2/DE	0.965	0.992
Native richness	C3/DE	1.052	0.971
Exotic richness	ABC1/C2	0.948	0.977
Exotic richness	ABC1/C3	0.756	0.128
Exotic richness	ABC1/DE	0.953	0.981
Exotic richness	C2/DE	1.005	1
Herb richness	C3/DE	1.279	0.115
